# An Intelligent Correlation Real-Time Analysis Method for the Mechanical Properties of Members in Super-Span Valve Hall Grid Structure Hoisting Process

**DOI:** 10.3390/s22218111

**Published:** 2022-10-23

**Authors:** Qinghe Zeng, Jin Liao, Xionghui Huang, Weihua Ming, Yi Gao, Cuiying Zhou, Zhen Liu

**Affiliations:** 1 China Southern Power Grid Ehv Power Transmission Company, Guangzhou 510275, China; 2 School of Civil Engineering, Sun Yat-sen University, No. 135 Xingangxilu, Guangzhou 510275, China; 3 Guangdong Engineering Research Centre for Major Infrastructure Safety, Guangzhou 510275, China

**Keywords:** super-span valve hall, hoisting process, mechanical properties of members, intelligent correlation, real-time analysis

## Abstract

The mechanical performance analysis of the members is the primary basis for evaluating the hoisting quality and safety of the valve hall grid structure. Ordinarily, manual analysis of monitoring data and on-site experience inspection are employed to structural judgment, but it is challenging to evaluate the correlation of the various members and the overall safety of a valve hall. In this paper, an intelligent correlation real-time analysis method based on a BPNN (Back Propagation Neural Network) for the mechanical properties of members is proposed to intelligently control the safety of valve hall grid structure hoisting. The correlation between the mechanical properties of multi-points in the grid structure is used to model the target measuring points. In addition, an intelligent real-time analysis system is used to manage and apply the mechanical property correlation and abnormality of members in real-time. Then, the model is applied to a super-span valve hall in South China, and the application effect is good. The mechanical property correlation model can accurately reflect the mechanical state of the valve hall grid structure hoisting process. Simultaneously, it can effectively pinpoint hidden dangers and locate risk members. It provides a new reference for the normal operation and maintenance of a super-span valve hall grid.

## 1. Introduction

The integral hoisting construction of a super-span valve hall grid structure involves complex system engineering, which affects the progress, safety, and quality of the whole valve hall project. Because of the weight of a steel structure, the process of hoisting has a great impact on the structural mechanics of the grid structure. Therefore, it is critical to monitor the mechanical properties of each member to ensure the safety and stability of super-span space grid structures [1,2,3]. Usually, a certain number of monitoring control points will be selected during the staged hoisting of the super-span valve hall grid structure in the converter station. The mechanical properties of several key members in the integral hoisting process are effectively monitored to ensure the safety of the valve hall grid structure [4,5]. The mechanical performance monitoring method, based on sensors used in the structural condition monitoring system, can only cover the limited range of the super-span valve hall. It cannot reflect the mechanical state of the whole grid structure itself. In addition, the current analysis of mechanical properties is mostly based on single-point model analysis and inspection. However, the stress and development of any monitoring point do not exist independently. It is affected by other monitoring points. Therefore, the key to ensuring the safety and stability of the valve hall construction is to use a low-cost and efficient intelligent real-time analysis method to evaluate the overall safety form in the process of grid hoisting.

At present, the monitoring system is generally configured for the grid steel structure of a converter station valve hall. It mainly collects a large amount of data for operation and maintenance personnel to judge and maintain. There are two methods to monitor and analyze the mechanical properties of the members during valve hall grid structure integral hoisting. The first is based on the structural derivation and design drawings. Scholars mainly estimate the change in mechanical properties of each member in the valve hall grid structure through numerical analysis. Clearly, this theoretical calculation method is simple and fast. However, because of the lack of relevant information, it is challenging to simulate the limitations of the real stress state under complex structural factors. The calculation error is large, sometimes even inaccurate [6,7,8,9,10,11]. The other method is to collect the mechanical data of each member on site according to various measuring instruments (e.g., optical fiber, stress gauge, and other mechanical sensors). The experts mainly analyze the change rules of their structural mechanics to evaluate the working state and safety form of the grid structure over time. This method is more accurate than the former method and can assess real-time changes in the structural mechanics of each member. The accuracy of the results is proportional to the number of measuring instruments employed on-site. However, the hoisting of a super-span valve hall grid structure has particular features. The complex environment and overlapping processes at the construction site easily lead to signal transmission failure and equipment failure. In addition, the relevant measuring instruments can only cover the limited range of the grid structure. Therefore, the processing and mining of limited data collected by contact sensors is still a major challenge [12,13,14,15,16,17]. To sum up, the mechanical property data of each member in the whole process of integral hoisting of a super-span valve hall grid structure are mainly obtained through manual measurement and real-time signal acquisition from on-site sensors. There are limitations such as data stream data interference and data processing failures. This causes problems such as low efficiency, high cost, and high risk of data monitoring and processing. More importantly, the single-measurement analysis and inspection is difficult to truly reflect the correlation between the members and the overall real-time safety form of the valve hall [18,19,20]. Therefore, it is necessary to choose an intelligent real-time analysis method with strong correlation, high efficiency, and low cost to evaluate the overall operational state of the valve hall grid during the lifting process. In particular, research on the intelligent correlation analysis of the mechanical properties of multiple measuring points is key to the overall operational status and monitoring assistance of the valve hall hoisting project.

In view of the above problems, this paper takes a super-span valve hall grid frame hoisting project in South China as the research object. We propose an intelligent correlation real-time analysis method based on a BPNN (Back Propagation Neural Network) for the mechanical properties of multiple measuring points. The correlation between the mechanical measurements of multiple measuring points in the grid space is used to model the target measuring points. In addition, the intelligent real-time analysis system is used to effectively manage and apply the mechanical property correlation and abnormality of each member in real time. The results show that the mechanical property correlation model established in this paper can more accurately reflect the mechanical state of the whole lifting process of the valve hall grid structure. Simultaneously, it can effectively excavate hidden dangers and locate members that are failing. It provides a new reference for the normal operation and maintenance of the super-span valve hall grid.

## 2. Materials and Methods

### 2.1. Intelligent Correlation Principle for Mechanical Properties of Multiple Measuring Points

For the super-span valve hall, because of its huge volume, the stress state of the structure itself needs to be considered during the construction and hoisting. Therefore, in the process of hoisting the valve hall grid structure, the analysis of the mechanical properties of the members is of great significance for evaluating the quality and safety of the project [21,22]. Monitoring the stress performance of the whole hoisting process of the super-span valve hall grid structure can help us to better grasp the stress situation of the structure and ensure smooth construction.

At present, the single-measuring-point model is widely used for establishing the mechanical monitoring model of the member, that is, to establish a monitoring model for the mechanical data of each monitoring point and identify its state through the mechanical state of each member at different times [23,24,25]. However, with this single-measurement analysis and inspection methods, it is difficult to determine the correlation between the members in the lifting process of the valve hall. It is also nearly impossible to truly evaluate the overall safety form of the valve hall.

In view of the aforementioned problems, we used the mechanical measurement sequence of multiple measuring points in space to jointly consider multiple measuring points. Through the correlation between the measuring points, the target measuring points were modeled. Based on a BPNN algorithm, a multiple-measuring-point mechanical property correlation model of the valve hall grid members was established.

The basic process of the multiple-measuring-point mechanical property correlation model of the member was as follows:

(1) The mechanical data monitoring network of a valve hall grid structure is set with *n* monitoring points. The mechanical values of *n* − 1 measuring points are used as variables, and the other measuring points are used as target values to build a model to predict unknown points. Specifically, the measured value of *P*_1_ is predicted by fitting the data of (*P*_2_, *P*_3_, …, *P*_*n*−1_, *P_n_*) measuring points.

(2) Similarly, the measured values of P_2_ are fitted and predicted with the data of (*P*_1_, *P*_3_, …, *P*_*n*−1_, *P_n_*) measuring points.

(3) By analogy, it is gradually extended to all monitoring points. The mechanical values of each member of the whole valve hall grid are solved. Namely, the measured value of *P_n_* is predicted by fitting the data of (*P*_1_, *P*_2_, …, *P*_*n*−2_, *P*_*n*−1_) measuring points.

(1) $$\left\{ \begin{array}{l} P_{1}=F\left(P_{1},P_{2},\ldots ,P_{i},\ldots ,P_{n-1},P_{n}\right)\\ P_{2}=F\left(P_{1},P_{3},\ldots ,P_{i},\ldots ,P_{n-1},P_{n}\right)\\ \ldots \\ P_{i}=F\left(P_{1},P_{2},\ldots ,P_{i-1},P_{i+1},\ldots ,P_{n}\right)\\ \ldots \\ P_{n-1}=F\left(P_{1},P_{2},\ldots ,P_{i},\ldots ,P_{n-2},P_{n}\right)\\ P_{n}=F\left(P_{1},P_{2},\ldots ,P_{i},\ldots ,P_{n-2},P_{n-1}\right) \end{array},\right.$$

where $P_{i}$ is the *i*-th monitoring point, 1≤i≤n; F( ) is the multi-measuring-point mechanical correlation function of the valve hall structural member; and *n* is the total amount of mechanical data of each monitoring point obtained at the project site.

Through the establishment of the multiple-measuring-point mechanical property correlation model of the valve hall grid members, the overall mechanical field of the valve hall in the hoisting process can be understood. In addition, the mechanical mechanism and stress variation law contained in the data of each monitoring point in the grid can be fully explored so as to find problems as early as possible and take countermeasures to eliminate hidden dangers.

### 2.2. Mechanical Property Correlation Model of Multiple Measuring Points

#### 2.2.1. Determination of Sample Space of the Model

As mentioned above, the single-measuring-point model needs to model different measuring points separately, without considering the relationship between measuring points. The time series mechanical model of a single measuring point cannot reveal the mechanical field of the valve hall in the hoisting process, and so does not fully reflect the overall situation. Therefore, we needed to establish the model sample space first, so as to aid the construction of the BPNN structure, which is a correlation model for the mechanical properties of multiple measuring points of valve hall grid structure members.

#### 2.2.2. BPNN Construction

**Figure 1:**
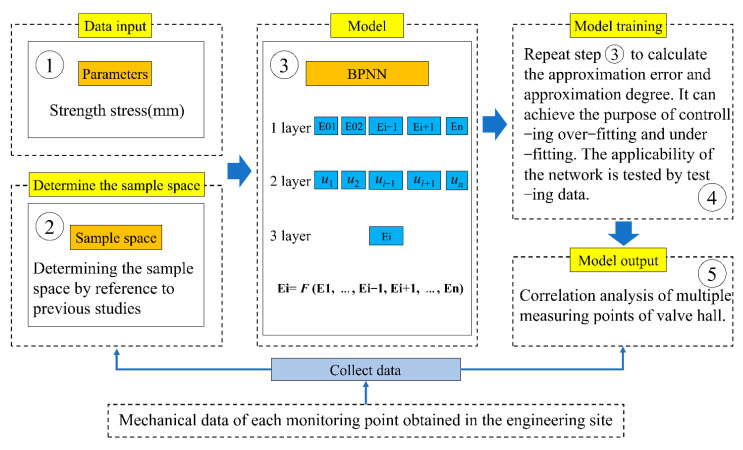
Research flow of intelligent analysis on mechanical properties of multiple measuring points of valve hall grid structure members based on a BPNN.

The research process of a BPNN for the multiple-measuring-point mechanical property correlation model of valve hall grid members is shown in Figure 1. Initially, the mechanical property data of each monitoring point of the valve hall grid members were obtained according to the on-site monitoring. Then, the mechanical data of *n* − 1 multiple measuring points were taken as the input variable of the BPNN, and the mechanical data of another measuring point were taken as a group of output variables. From this, *n* training sample spaces of the BPNN were constructed. We input the data parameters into the BPNN for training. Each training sample space was iterated many times to make the convergence error reach the set value and determine the parameters of the neural network. After the model training, we calculated the relative error between the measured value and the predicted value of the mechanical data of each member of the valve hall grid, and then checked the correctness of the model. Finally, the correlation of mechanical properties of multiple measuring points and the overall safety form results of the valve hall grid hoisting project were obtained through the model.

**Figure 2:**
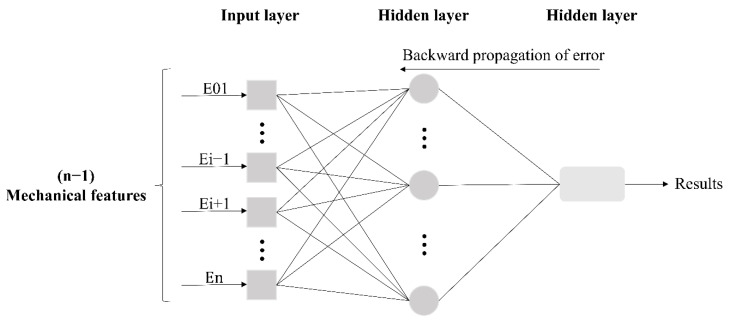
The intelligent analysis of a BPNN in the correlation of mechanical properties of multiple measuring points of valve hall members.

This paper used a three-layer BPNN to learn and predict the samples (Figure 2):

(2) Ei=F(E01, E02, …, Ei−1, Ei+1, …, En),

where Ei is the mechanical data of the *i*-th monitoring point, 1≤i≤n; F( ) is the multi-measuring-point mechanical correlation function of the valve hall structural member; and *n* is the total amount of mechanical data of each monitoring point obtained at the project site.

Because it is limited to a one-dimensional nonlinear time series process, the output layer only needs one node. In this way, the mechanical data of *n* − 1 measuring points and the mechanical data of another measuring point are taken as target values to build a model to predict the unknown points.

The specific expression of the multiple-measuring-point mechanical property correlation model of the member is shown in Equations (3)–(6):

(3) $$\alpha_{h}=\sum_{i=1}^{M}v_{ih}x_{i}+r_{h}$$

(4) $$b_{h}=f\left(\alpha_{h}\right)$$

(5) $$y_{j}=\sum_{h=1}^{q}w_{hj}b_{h}+\theta_{j}$$

(6) $$f\left(x\right)=\frac{1}{1+e^{-x}},$$

where *M* is the number of nodes in the input layer; *v_ih_* is the *M* dimension weight vector; $x_{i}$(*i* = 1, 2, ……, *M*) is the column vector value corresponding to the sequence; *h* is the number of hidden layer nodes; $r_{h}$ is the threshold parameter of the neural network from the input layer to the hidden layer; *q* is the number of nodes in the output layer; $w_{hj}$ is the weight parameter of the neural network from the hidden layer to the output layer; $\theta_{j}$ is the threshold function from the hidden layer to the output layer of the neural network; and *y_j_* (*j* = 1) is the predicted output value of the monitoring point corresponding to the sequence.

### 2.3. An Intelligent Real-Time Analysis System for the Mechanical Properties of Grid Members

**Figure 3:**
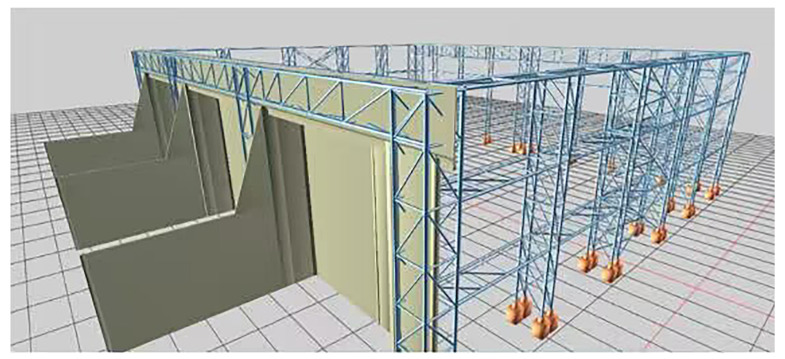
An intelligent real-time analysis system for the mechanical properties of members in the whole process of the hoisting of a large-span valve hall space grid.

**Figure 4:**
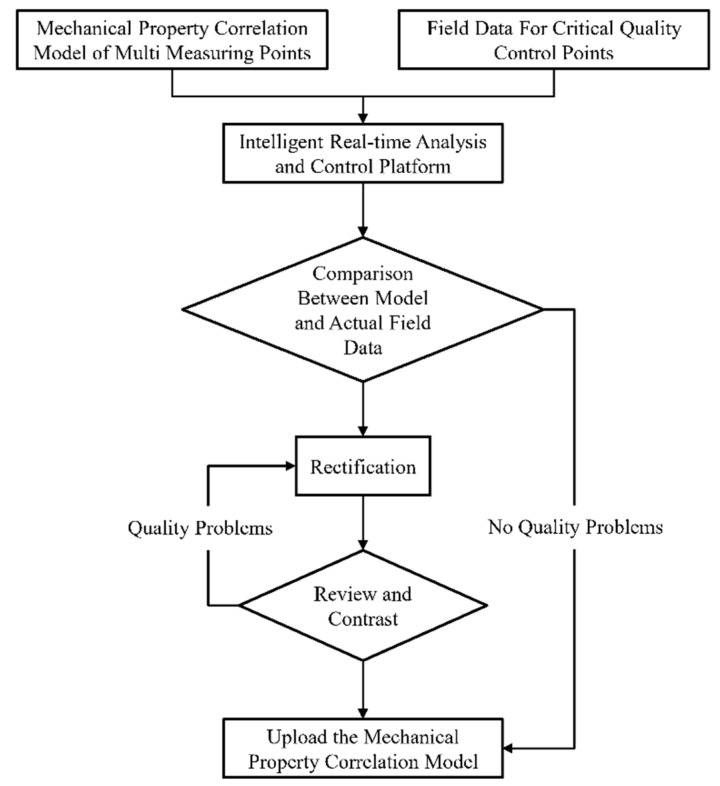
Intelligent real-time analysis and control process.

Combined with our team’s previous research results and practical engineering experience [26,27], the multidisciplinary data management and application technology of valve hall engineering based on microservice architecture can improve the management efficiency of engineering data. Through the multiple-measuring-point mechanical property correlation model of the members, the correlation between the members in the grid structure of the valve hall project is established without increasing the amount of model data required. The correlation effect of the model is evaluated by the determination coefficient in Equation (7). Based on this, the intelligent real-time analysis system (Figure 3) for the mechanical properties of members is established to effectively manage and apply the mechanical properties of each member in real-time. In addition, we can click a steel node or steel member in the model through the intelligent real-time analysis system to obtain its specific state. By displaying and analyzing the three-dimensional model, the construction progress of grid frame hoisting can be controlled and decided. The main intelligent real-time analysis and control processes are shown in Figure 4.

(7) $$R^{2}=\frac{{\left(n\sum_{i=1}^{n}\hat{m_{i}}\cdot m_{i}-\sum_{i=1}^{n}\hat{m_{i}}\cdot \sum_{i=1}^{n}m_{i}\right)}^{2}}{(n\sum_{i=1}^{n}{\hat{m_{i}}}^{2}-\left(\sum_{i=1}^{n}\hat{m_{i}}{)}^{2}\right)\left(n\sum_{i=1}^{n}m_{i}{}^{2}-{\left(\sum_{i=1}^{n}m_{i}\right)}^{2}\right)},$$

where *n* is the number of samples, $\hat{m_{i}}$ is the predicted value of the i-th sample, and $m_{i}$ is the true value of the nth sample. The decisive coefficient $R^{2}$ is within [0,1]. The closer $R^{2}$ is to 1, the better the performance of the model.

**Figure 5:**
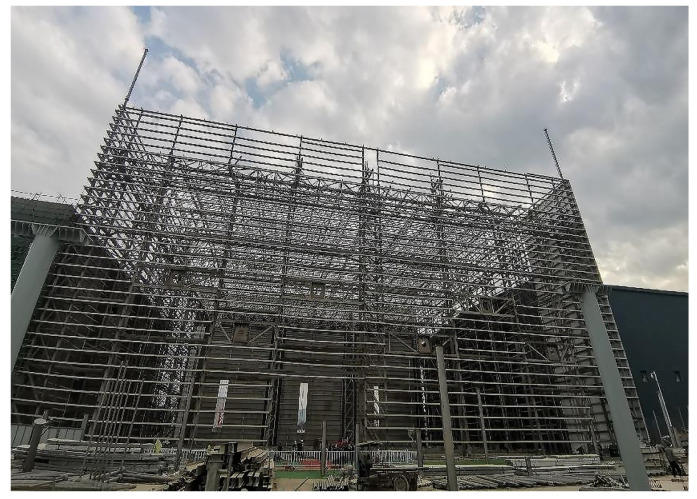
Converter station steel structure site.

**Figure 6:**
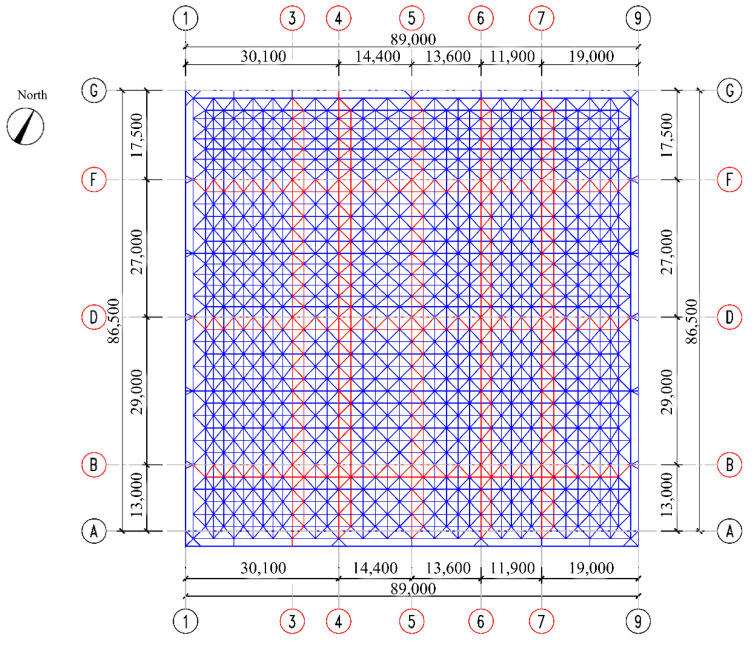
Layout of monitoring points. (Note: the red part of the figure is the monitoring area.)

**Figure 7:**
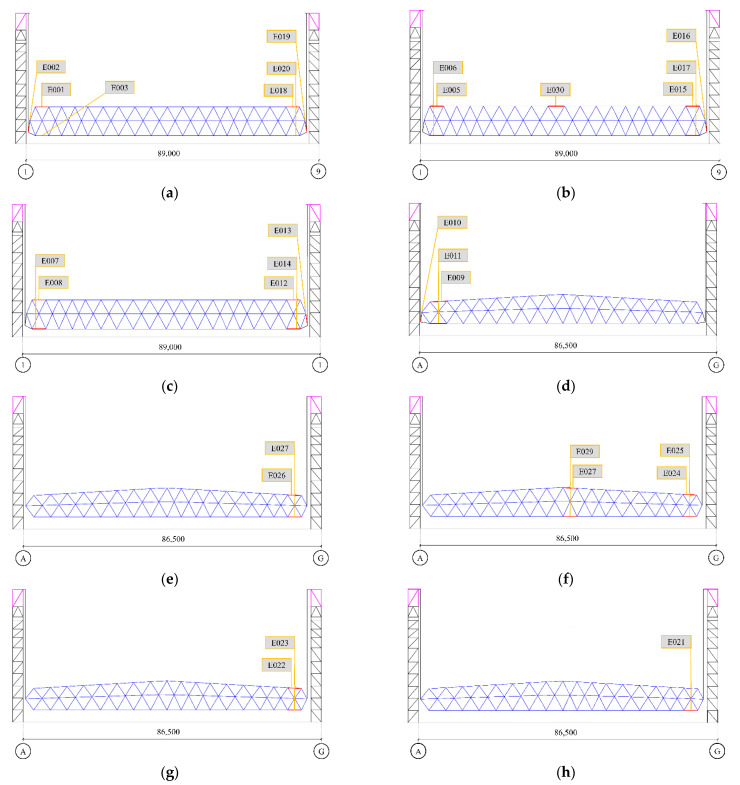
Distribution of monitoring points in the section: (**a**) F-axis section; (**b**) D-axis section; (**c**) B-axis section; (**d**) 3-axis section; (**e**) 4-axis section; (**f**) 5-axis section; (**g**) 6-axis section; (**h**) 7-axis section. (Note: E stands for monitoring point number letter.)

To test the performance of the above multiple-measuring-point mechanical property correlation model and the application effect of the intelligent real-time analysis system, the high-end valve hall of a converter station in South China was used as the test bench in this paper (Figure 5). The high-end valve hall was a steel structure with a maximum installation elevation of +43.971 m. The grid structure of a high-end valve hall was processed and pre-arched, with a value of 0.1 m. The lifting area was a roof grid structure, which adopts hydraulic lifting. The lifting range was between the 1~9 axis and the A~G axis of the structure. The maximum span of the structure was 89.0 m, and its height was 8.471 m. The lifting height was about 35.5 m, and the lifting weight was 733 t. Stress observations were carried out at the four stages of member installation, hoisting 0.5 m, hoisting 15 m, and hoisting to the top before hoisting the valve hall. As shown in Figure 6 and Figure 7, the monitoring points were set at axis 3~7 and axis B~F of the valve hall structure. Figure 6 is the vertical view of the monitoring point layout, while Figure 7 represents the sectional view. A total of 30 monitoring points were arranged along the axis of the valve hall, and the monitoring point numbers were E001–E030.

**Figure 8:**
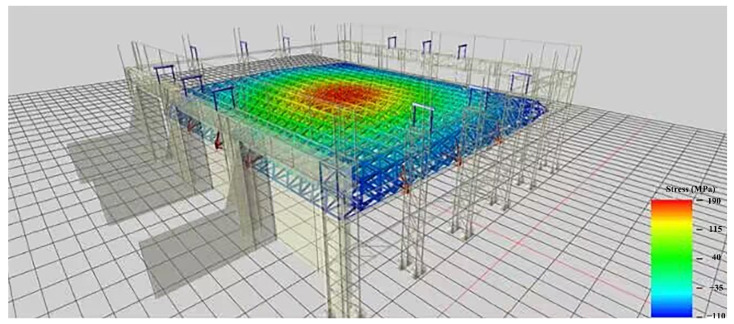
Monitoring data collection of an intelligent real-time analysis system.

**Figure 9:**
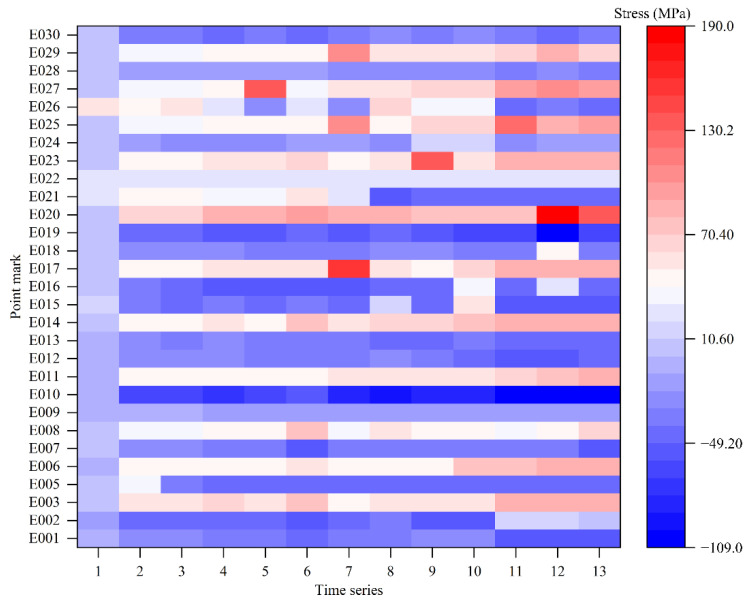
Monitoring data of each member.

The monitoring points were monitored along with the gradual hoisting of the valve hall grid structure. To ensure the reliability and continuity of the monitoring data, the damage point E004 was eliminated in advance. The intelligent real-time analysis system, based on the mechanical properties of the members in the whole hoisting process of the grid structure, collected the monitoring data (Figure 8). This test was based on 13 time series data points from four stages of member stress monitoring data obtained at key monitoring points (E001, E002, E003, and E005–E030) (Figure 9). Figure 9 shows the change in stress values at each monitoring point in the time series. We can measure the stress value by color. The monitoring stress of the series (E002, E003, and E005–E030) was used as the input data to predict the stress of E001. Then, the stress of the series (E001, E003, and E005–E030) was used as the input data to predict the stress of E002. The process was completed by analogy until 29 model training samples were established.

## 3. Results and Discussion

### 3.1. Multiple-Measuring-Point Mechanical Property Associated Sample Training Test of Member

**Figure 10:**
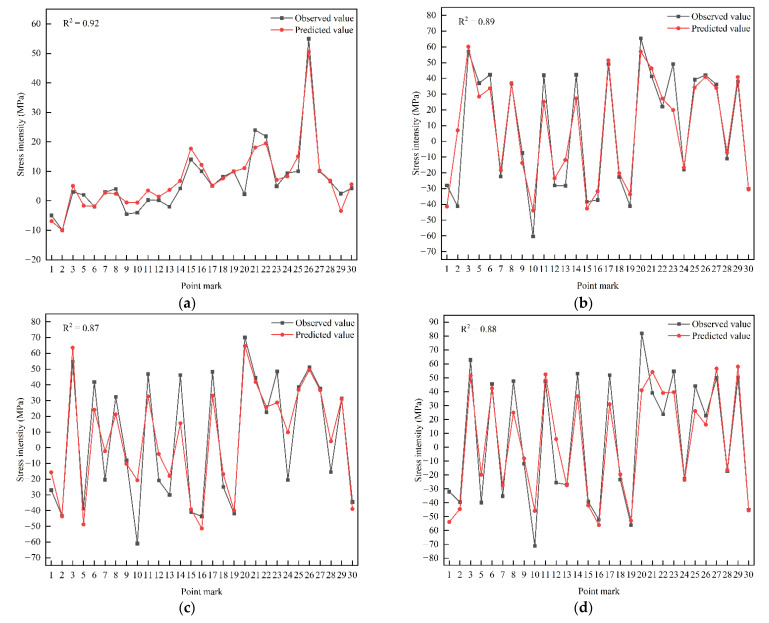

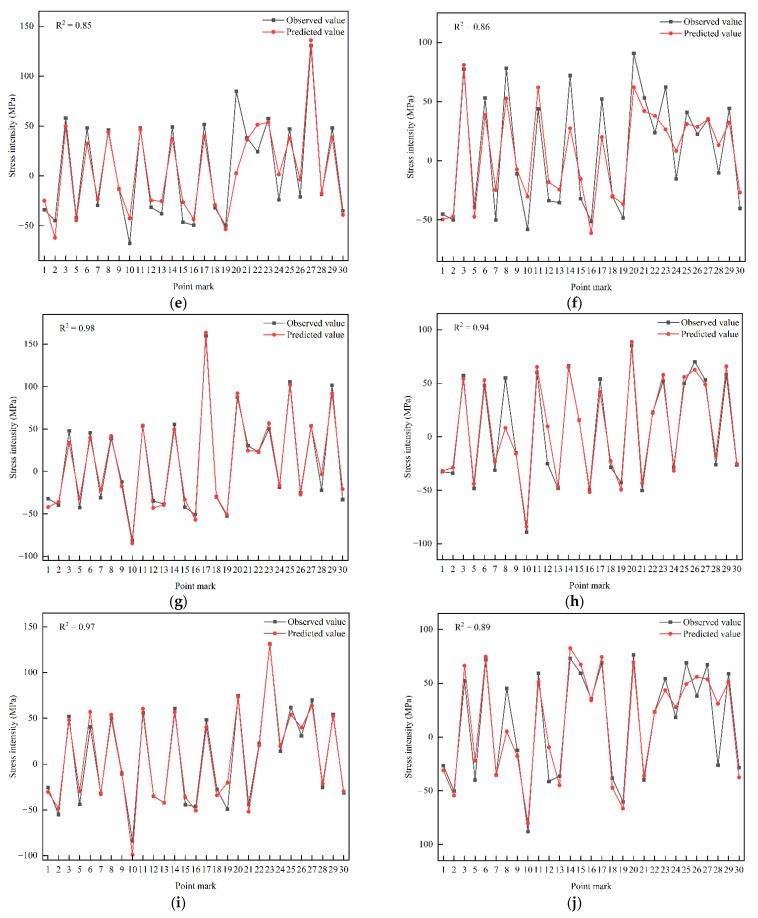

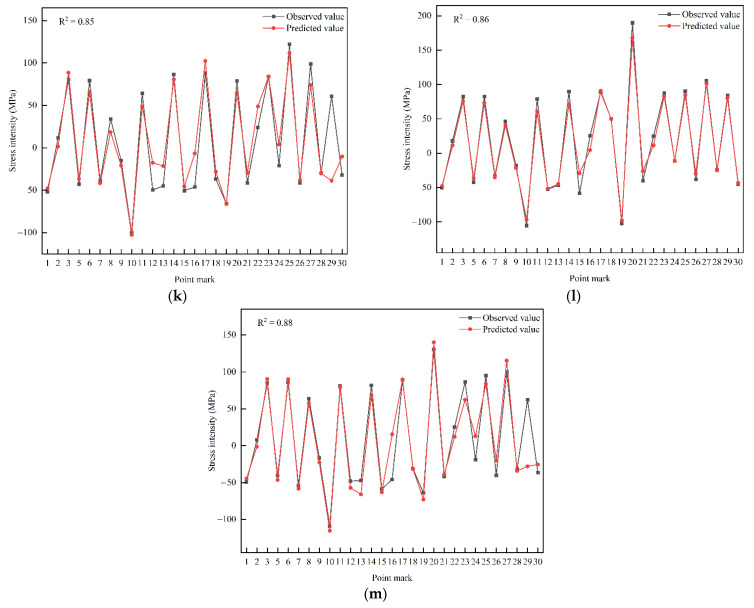
Correlative prediction output diagram of mechanical properties of multiple stages and multiple measuring points for valve hall hoisting. (**a**) Time series 1; (**b**) time series 2; (**c**) time series 3; (**d**) time series 4; (**e**) time series 5; (**f**) time series 6; (**g**) time series 7; (**h**) time series 8; (**i**) time series 9; (**j**) time series 10; (**k**) time series 11; (**l**) time series 12; (**m**) time series 13.

According to the BPNN structure constructed in Section 2.2, the number of input layer units is 28, and the number of output layer units is 1. We studied and trained the mechanical property correlation model of the multiple measuring points of the member through the following steps:

(1) Initialize BPNN. We initialized the neural network parameters with the data in Figure 9 as training and test samples. Then, we normalized the sample data.

(2) Training BPNN. We selected 90% of the sample data to train the BPNN until the end of the network training. BPNN converges after learning and training. That is to say, the mechanical property correlation model of multiple measuring points of the member based on BPNN was obtained.

(3) Check the BPNN. We randomly selected 10% sample data for inspection. The trained BPNN was used to predict the sample data, and the prediction output diagram was obtained (Figure 10). Finally, Equation (7) was used to evaluate the mechanical property correlation model of multiple measuring points.

It can be seen from the curve comparison that the curve change rules of the training results are basically consistent with the measured curves (Figure 10), including the different laws for measuring stress in different periods. This shows that the selection of the input layer factor and the mechanical property correlation model of the multiple measuring points in this paper achieved the expected good results (*R*^2^ = 0.85–0.98). It indicates that the multiple-measuring-point mechanical property correlation model can fully explore the mechanical correlation mechanism and mechanical change rules contained in each member and has high prediction and fitting accuracy.

The input space was set as x and the output space as y. It was assumed that the relationship between x and y can be described by an unknown true mapping function *y* = *g*(*x*). The goal of building a neural network is to find a model to approximate the true mapping function [28,29]. Therefore, the correlation between the parameters of the output factor and the input factor can be obtained by deriving the training parameters of the neural network. That is, the specific characterization of the mechanical correlation model of each measuring point member is obtained through Equations (3)–(6), where *M* = 28; *v_ih_* is the *M* dimension weight vector; $x_{i}$ (*i* = 1, 2, ……, *M*) are column vector values corresponding to sequences (E02, E03, E05–E30), (E001, E003, E005–E030), …, and (E001, E002, E003, E005–E029), respectively; *h* is the number of hidden layer nodes, 12; $r_{h}$ is the threshold parameter of the neural network from the input layer to the hidden layer; *q* is the number of nodes in the output layer, *q* = 1; $w_{hj}$ is the weight parameter of the neural network from the hidden layer to the output layer; $\theta_{j}$ is the threshold function from the hidden layer to the output layer of the neural network; and *y_j_* (*j* = 1) corresponds to the prediction outputs E001, E002, E003, E004, …, E030 of the monitoring points corresponding to sequences (E002, E003, E005–E030) and (E001, E003, E003, E005–E029).

Taking the prediction and analysis of the mechanical property data of each monitoring point in the first time series of the member installation stage as an example, the following values (*v_ih_*, $r_{h}$, $w_{hj}$, $\theta_{j}$) were obtained:

$$v_{ih}(28\times 9)=[\begin{array}{ccccccccc} -0.023 & -0.358 & 0.078 & 0.322 & -0.063 & 0.039 & -0.120 & -0.506 & 0.490\\ -0.414 & -0.393 & -0.077 & 0.131 & 0.501 & 0.409 & 0.400 & -0.166 & 0.336\\ \ldots & \ldots & \ldots & \ldots & \ldots & \ldots & \ldots & \ldots & \ldots \\ \ldots & \ldots & \ldots & \ldots & \ldots & \ldots & \ldots & \ldots & \ldots \\ -0.284 & 0.203 & 0.508 & -0.542 & 0.567 & 0.253 & 0.509 & -0.526 & -0.387\\ 0.112 & -0.126 & -0.267 & -0.278 & 0.175 & -0.409 & 0.164 & -0.020 & -0.279 \end{array}]\;r_{h}={\left[0.044\;0.007\;-0.400\;-0.191\;-0.332\;-0.006\;-0.548\;-0.358\;0.652\right]}^{T}\;w_{hj}=\left[0.840\;0.476\;0.487\;0.692\;-0.541\;0.093\;-0.455\;0.510\;-0.151\right]\;\theta_{j}={\left[0.077\right]}^{T}$$

### 3.2. Identification of Abnormal Members with Multiple Measuring Points

#### 3.2.1. Identification Principle of Abnormal Members with Multiple Measuring Points

Combined with the intelligent real-time analysis system of the mechanical properties of the member and BPNN, the abnormal data of the mechanical properties of each member can be identified through on-site observation and numerical simulation.

Neural networks are generally complex and refer to the flow of data between the original input of a sample and the output target through multiple nonlinear components. Because each component processes the information and in turn influences subsequent components, we end up with an output where we do not know how much each component contributed. BPNN is a model that better solves the contribution distribution problem [30,31]. Based on the mechanical correlation and mutual influence between the members established above, the field observation and numerical simulation of monitoring points are used as influence factors to participate in the prediction modeling process of the benchmark. In this paper, E030, in the middle of the grid, was selected as the reference point, and the time domain was taken as the influence element. Finally, the influence contribution sequence of each monitoring point to E030 mechanics was sorted.

Since the numerical simulation only considers the three stages of hoisting before, during, and to the top, the time domain of the three hoisting stages of each monitoring point was taken as the input eigenvector. At the same time, the time domain of monitoring point E030 was taken as the output feature vector, and the contribution sequence of each monitoring point to the impact of E030 mechanics was obtained and sorted.

Finally, the contribution of each member node to the mechanical impact of monitoring point E030 under normal and abnormal conditions was obtained. This served to compare the contribution of theoretical conditions (mechanical data under numerical simulation) and actual conditions (mechanical data under on-site monitoring) to the mechanical properties of E030.

#### 3.2.2. Training Test Analysis of Abnormal Data Samples of Multiple-Measuring-Point Mechanical Members

**Figure 11:**
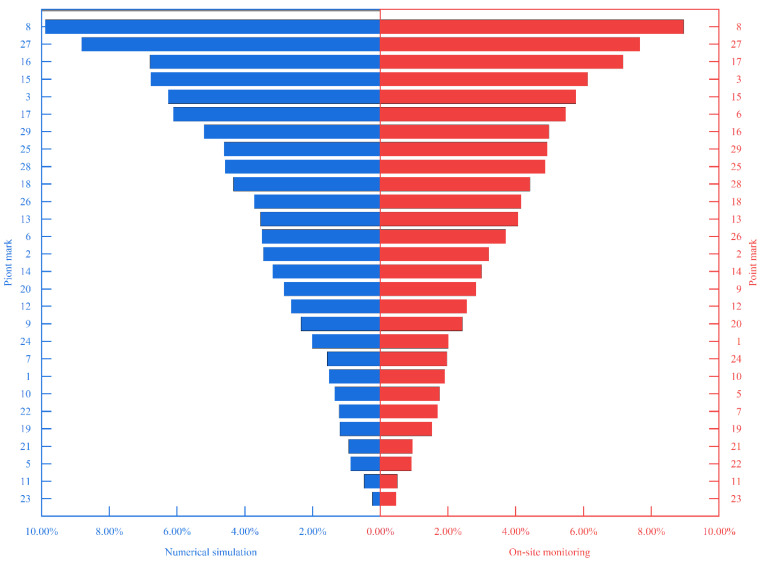
Contribution series of monitoring data and numerical simulation data on mechanical properties of E030 based on time domain.

According to the BPNN structure constructed in Section 2.2, the number of input layer units was 28, and the number of output layer units was 1. We learned and trained the monitoring data and numerical simulation data samples of the mechanical properties of multiple-measuring-point mechanical members through the following steps:

(1) Initialize BPNN. Based on the monitoring data and numerical simulation data samples of the mechanical properties of the grid structure, each monitoring point except E030 was taken as the input vector. Then, we predicted the time-lapse mechanical value of monitoring point E030. The neural network parameters were initialized with the sample space as training and test samples. Then, the sample data were normalized.

(2) Training BPNN. We selected 90% of the sample data to train the BPNN until the end of the network training. BPNN converges after learning and training. That is to say, based on BPNN, a correlated intelligent analysis model for the mechanical properties of multiple measuring points of valve hall grid members was obtained.

(3) Check the BPNN. We randomly select 10% sample data for inspection. The trained BPNN was used to predict the sample data.

(4) Based on Equations (3)–(6), the relationship between the parameters of the output factor and the input factor was obtained by deriving the training parameters of the neural network from the mechanical data of the monitoring point E030 in the above time domain. In the case of on-site monitoring and numerical simulation, the contribution sequence of the influence of the time domain on the mechanical properties of E030 was obtained (Figure 11).

**Figure 12:**
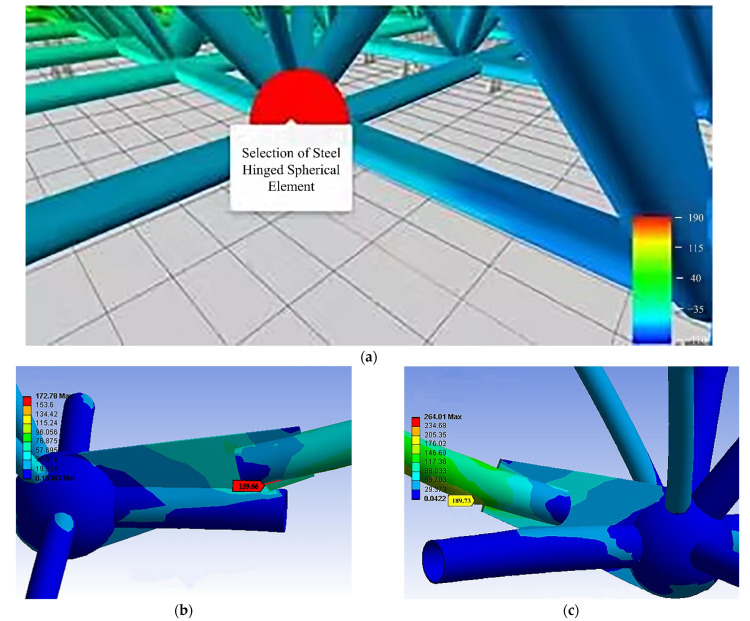
(**a**) Schematic diagram for the selection of the steel hinged spherical element; (**b**) stress inspection of member 17; (**c**) stress inspection of member 20.

Comparing the field monitoring and numerical simulation, we took the time domain as the contribution sequence of the influence factors on the mechanical properties of E030. It can be seen that the sequence of members numbered 17, 20, 6, and 7 varied greatly (Figure 11). Therefore, there is a risk of failure of these members. Through later troubleshooting of the intelligent real-time analysis system for the mechanical properties of the member (Figure 12), the stresses of members 17 and 20 were seen to exceed the first-level limit of steel stress (150 MPa). However, the third-level limit (270 MPa) was unreached, so they were still in a safe state. Among them, members 6 and 7 were located at one of the lifting points, which were disturbed during the lifting process and caused abnormalities.

#### 3.2.3. Sample Evaluation of Abnormal Mechanical Data of Grid Members

Chaos is usually used to investigate the distribution of the elements in a set. We can measure the degree of confusion by the total number in reverse order. This paper evaluated the abnormal data of grid structure mechanics through the degree of confusion [32,33].

The influence contribution sequence of numerical simulation data on mechanical properties of E030 is based on the theoretical conditions. The arrangement of its contribution sequence must be the law obtained when there is no abnormality in each member. In fact, in the process of member installation and hoisting, the valve hall grid structure is affected by the deviation of member installation position, external load, and other factors. The original law of the contribution sequence of the influence on the mechanical properties of E030 obtained from the monitoring data of each member is destroyed and confused, and the degree of confusion must increase.

Therefore, CD (confusion degree) is defined as the chaos degree. ${\mathrm{CD}}_{1}$ is the chaos degree under the numerical simulation sequence. ${\mathrm{CD}}_{2}$ is the degree of confusion under the on-site monitoring sequence during the hoisting construction of the valve hall.

We obtained the following values: ${\mathrm{CD}}_{1}=47$, ${\mathrm{CD}}_{2}=44$, Amplitude of confusion $\left|\frac{{\mathrm{CD}}_{2}-{\mathrm{CD}}_{1}}{{\mathrm{CD}}_{1}}\right|\times 100\%=6.38\%$. The increase in confusion was small, which indicates that, although a few members in the valve hall grid structure exceeded the first-level limit, it was still in a relatively safe state as a whole. Therefore, we can make a qualitative evaluation of the overall safety pattern of the valve hall by the degree of confusion.

## 4. Conclusions

(1) In this paper, an intelligent correlation real-time analysis method based on a BPNN for the mechanical properties of multiple measuring points was proposed to intelligently control the safety form of the valve hall grid structure whole hoisting process. It solved the problems of low efficiency, high cost, high risk, and weak correlation of single-measurement analysis and inspection.

(2) Based on the BPNN algorithm, this paper used the correlation between the mechanical measurements of multiple measuring points in the grid space to model the target measuring points. The model achieved the expected good results (*R*^2^ = 0.85–0.98). This provides an effective new method for the intelligent analysis of the mechanical properties of grid members.

(3) In this paper, a multiple-measuring-point mechanical property correlation model and intelligent real-time analysis system of grid members were established on the time–space scale. They can effectively discover hidden dangers and locate faults that are occurring, and more accurately reflect the overall mechanical state of the valve hall, providing a new reference for the normal operation and maintenance of a super-span valve hall grid.

## Data Availability

Not applicable.
